# Change in total lesion PSMA (TLP) during [^177^Lu]Lu-PSMA-617 radioligand therapy predicts overall survival in patients with mCRPC: monocentric evaluation of a prospective registry

**DOI:** 10.1007/s00259-023-06476-x

**Published:** 2023-10-27

**Authors:** Caroline Burgard, Connor Hein, Arne Blickle, Mark Bartholomä, Stephan Maus, Sven Petto, Andrea Schaefer-Schuler, Samer Ezziddin, Florian Rosar

**Affiliations:** Department of Nuclear Medicine, Saarland University – Medical Center, Kirrberger Str. 100, Geb. 50, D-66421 Homburg, Germany

**Keywords:** Total lesion PSMA, Radioligand therapy, Metastatic castration-resistant prostate cancer, Survival

## Abstract

**Purpose:**

This study investigates imaging response of [^177^Lu]Lu-PSMA-617 radioligand therapy (RLT) based on the whole-body parameter total lesion PSMA (TLP), derived by PSMA-PET/CT and reflecting the total tumor burden, in patients with metastatic castration-resistant prostate cancer (mCRPC) enrolled in a prospective registry (NCT 04833517).

**Methods:**

A total of *n* = 102 mCRPC patients received a [^68^Ga]Ga-PSMA-11 PET/CT at baseline and after two cycles of PSMA-RLT, in which TLP was measured by using a semi-automated tumor segmentation. TLP was defined as the summed products of volume and uptake (∑ Volume × SUV_mean_) of all tumor lesions. The Kaplan-Meier method was used to determine the most appropriate ∆TLP thresholds for classification into partial remission (PR), stable disease (SD), and progressive disease (PD) regarding overall survival (OS). Furthermore, we analyzed criteria that are also frequently used in established response frameworks, such as the occurrence of new metastases as independent criterion (I) or in combination with change in tumor burden (II), and the change in PSA serum value (III).

**Results:**

For the ∆TLP thresholds −30%/+30% (and also for higher thresholds, −40%/+40% or −50%/+50%), significant differences between all three response categories became apparent (PR/PD: *p* = 0.001; PR/SD: *p* = 0.001; SD/PD: *p* = 0.018). Including the development of new metastases as independent criterion of PD, there was no significant difference in OS between SD and PD (*p* = 0.455), neither when applied in combination with TLP (*p* = 0.191). Similarly, significant differentiation between SD and PD was not achieved by PSA serum value (*p* = 0.973).

**Conclusion:**

In the largest monocentric study to date, TLP is shown to be a qualified prognostic biomarker, applying ∆TLP thresholds of −30%/+30%. It significantly differentiated between PR, SD, and PD, whereas other response criteria did not differentiate SD vs. PD. Using TLP, the development of new metastases is not a required information for predicting OS.

## Background

On a global scale, prostate cancer (PC) is ranked second of the most abundant malignancies. As age is one of the prominent risk factors, especially older men are at high risk of developing PC [1]. While survival expectancy is generally high in patients with early-stage PC, prognosis is poor in such with metastatic castration-resistant prostate cancer (mCRPC) [2–4]. This progressive form of PC is characterized by disease progression following surgical or pharmaceutical castration. Established therapeutic approaches in treatment of mCRPC include chemotherapy [5, 6], novel androgen axis drugs (NAAD) [7, 8] and PARP-inhibitors [9]. However, if the disease progresses despite prior treatment, radioligand therapy (RLT) is a promising option that has been recently approved by the US Food and Drug Administration (FDA) and European Medicines Agency (EMA). RLT targets the prostate specific membrane antigen (PSMA), a transmembrane glycoprotein which is overexpressed on mCRPC cells [10, 11]. PSMA-targeted RLT using [^177^Lu]Lu-PSMA-617 has been shown to be effective in various retrospective studies [12–15] as well as in phase II [16, 17] and phase III trials [18] with low side effects while reducing tumor burden. [^177^Lu]Lu-PSMA-617 RLT is considered effective especially for patients with high PSMA expression in tumor tissue [19]. In this respect, PSMA-targeted PET/CT, e.g., [^68^Ga]Ga-PSMA-11 PET/CT, is used as a staging and screening method to evaluate PSMA expression for PSMA-RLT [20]. Up to now, prostate specific antigen (PSA) is commonly used as an easily accessible biomarker for therapy response in RLT [21]. However, quantitative molecular imaging parameters derived from PSMA-PET/CT may be more favorable biomarkers. An appropriate assessment tool for therapy response is essential for further treatment decisions and has to meet several requirements. Specificity and reliability are important; additionally, measurement of the marker should blend into context of clinical routine. Previously proposed approaches for evaluating course of disease include quantitative measurement of target lesions, e.g., by the standardized uptake value (SUV) or SUV tumor-to-liver ratio [22, 23]. Alternatively, measurement of whole-body parameters can be applied, e.g., assessing the total molecular tumor volume [24, 25]. A promising biomarker that was addressed in a previous study by our group in a smaller cohort of patients, combining both uptake and volume of whole-body tumor burden, is the total lesion PSMA (TLP) [26], which is similar to the established total lesion glycolysis (TLG) in [^18^F]FDG PET/CT [27, 28]. This retrospective study investigates imaging response of [^177^Lu]Lu-PSMA-617 radioligand therapy based on TLP using a larger representative cohort in a real-world setting of patients enrolled in a prospective register, REALITY study, NCT 04833517.

## Materials and methods

### Patient population

This study analyzed *n* = 102 patients of the “*prospective registry to assess outcome and toxicity of targeted radionuclide therapy in patients with mCRPC in clinical routine (REALITY Study)*”, NCT04833517. Patients included were men with diagnosed late-stage or end-stage prostate cancer receiving [^177^Lu]Lu-PSMA-617 radioligand therapy at our institution between 1 January 2016, and 14 February 2023. All patients received multiple prior therapies, including chemotherapy, NAAD, or androgen deprivation therapy (ADT). Summarized patient characteristics can be seen in Table 1.

**Table 1:** Patient characteristics

Patient characteristics	Value
Age	
Median in [years], (range)	72 (48–88)
Age ≥ 65 years, *n* (%)	82 (80.4)
Age < 65 years, *n* (%)	20 (19.6)
ALP, in [U/L]	
Median (range)	109 (22–1753)
Hemoglobin, in [g/dL]	
Median (range)	12 (6–16)
< 13 g/dL, *n* (%)	70 (68.6)
ECOG performance status, *n* (%)	
0	29 (28.4)
1	51 (50.0)
≥2	22 (21.6)
Sites of metastases, *n* (%)	
Bone	93 (91.2)
Lymph node	79 (77.5)
Liver	17 (16.7)
Other	29 (28.4)
Prior therapies, *n* (%)	
Prostatectomy	51 (50.0)
Radiation	63 (61.8)
ADT	102 (100)
NAAD	97 (95.1)
Abiraterone	74 (72.6)
Enzalutamide	84 (82.4)
Abiraterone and enzalutamide	61 (59.8)
Chemotherapy	67 (65.7)
Docetaxel	66 (64.7)
Cabazitaxel	28 (27.5)
Docetaxel and cabazitaxel	27 (26.5)
[^223^Ra]Ra-dichloride	18 (17.7)
Other	22 (21.6)
PSA at baseline, in [ng/mL]	
Median (range)	130 (2.9–9579)
PSMA-RLT cycles	
Median (range)	5 (2–18)
Activity of ^177^Lu first cycle, in [GBq]	
Median (range)	7.7 (1.1–11.6)
≥ 5 GBq	98
<5 GBq	4
Activity of ^177^Lu second cycle, in [GBq]	
Median (range)	7.1 (1.5–9.9)
≥5 GBq	91
<5 GBq	11

*ADT* androgen deprivation therapy, *ALP* alkaline phosphatase, *ECOG* Eastern Cooperative Oncology Group, *NAAD* novel androgen axis drugs, *PSA* prostate specific antigen

**Fig. 1:**
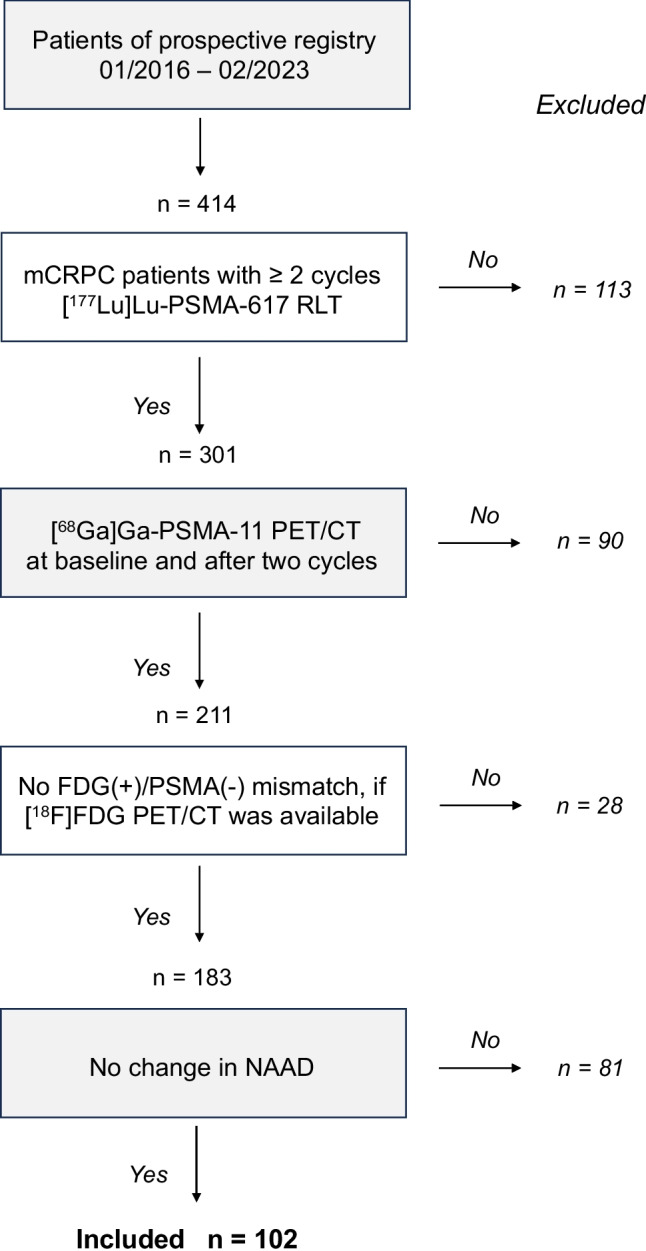
Flowchart presenting the systematics of patient selection

According to inclusion criteria, patients had to be diagnosed with mCRCP, had not received previous RLT, and had to undergo [^68^Ga]Ga-PSMA-11 PET/CT for staging, and again for follow-up after two cycles of [^177^Lu]Lu-PSMA-617 RLT. If [^18^F]FDG PET/CT was performed additionally, patients with mismatch findings in [^18^F]FDG PET/CT and [^68^Ga]Ga-PSMA-11 PET/CT were excluded. In addition, patients who received a change in NAAD medication during the study period were excluded to avoid artificial changes in PSMA expression. This refers to patients who discontinued NAAD medication or were newly prescribed in-between the two RLT cycles Fig. 1.

Informed consent was obtained from all patients involved in this study and was conducted according to the guidelines of the Declaration of Helsinki. PSMA-RLT was performed in consensus to the German Pharmaceutical Act §13 (2b). The analysis was approved by the local Institutional Review Board (ethics committee permission number 140/17).

### Imaging and treatment details

Each patient received a [^68^Ga]Ga-PSMA-11 PET/CT 14 ± 13 days before the first [^177^Lu]Lu-PSMA-617 RLT cycle was administered. The follow-up [^68^Ga]Ga-PSMA-11 PET/CT was performed 37 ± 10 days after the second cycle. Median activity was 132.5 MBq (range 77–195 MBq) for the staging PET/CT, and 128 MBq (range 95–250 MBq) for the follow-up scan. Blood samples were collected right before the intravenous injection of the tracer. Samples were tested for quantitative values of PSA, full blood count, and alkaline phosphatase. Administration of [^68^Ga]Ga-PSMA-11 was followed by 500 mL infusion of 0.9% NaCl. ^68^Ga was provided via Eckert & Ziegler Strahlen- und Medizintechnik AG (Berlin, Germany) using a ^68^Ga/^68^Ge generator. The PSMA ligand PSMA-11 was obtained from ABX advanced biochemical compounds GmbH (Radeberg, Germany). In accordance with the guidelines of prostate cancer imaging [29], time period between injection and imaging was 60 min. PET/CT scans were performed using a Biograph 40 mCT PET/CT scanner (Siemens Medical Solutions, Knoxville, TN, USA). The acquisition time was 3 min/bed position, the slice thickness was 3.00 mm, and an extended field of view of 21.4 cm (TrueV) was used. For attenuation correction and anatomic localization, low-dose CT was acquired with an X-ray tube voltage of 120 keV and modulation of the tube current using CARE Dose4D with a reference tube current of 50 mAs. With an increment of 3.0 mm and a slice thickness of 5.00 mm, the CT scans were reconstructed with a 512 × 512 matrix. Using a three-dimensional OSEM algorithm with 3 iterations, 24 subsets, Gaussian filtering, and a slice thickness of 5.00 mm, PET reconstruction was performed. Random correction, scatter correction, decay correction, and attenuation correction were implemented.

Every patient included in this study received two cycles of [^177^Lu]Lu-PSMA-617 RLT. The median activity of the first cycle was 7.7 GBq (range 1.1–11.6 GBq). For the second cycle, a median activity of 7.1 GBq (range 1.5–9.9 GBq) was applied. The administered activities were adjusted to patient’s specific characteristics such as tumor progression, distribution and extent of tumor burden, body surface, renal function, or blood cell count. Low activity (<5 GBq) was administered due to pre-existing terminal impairment of kidney function. The time interval between both RLT cycles was 40 ± 10 days. After two cycles of [^177^Lu]Lu-PSMA-617 RLT, the median cumulative activity was 14.9 GBq (range 2.6–19.4 GBq). The administered [^177^Lu]Lu-PSMA-617 was synthesized in accordance with standard procedures as described by Kratochwil et al. [30]. PSMA-617 was obtained from ABX advanced biochemical compounds GmbH (Radeberg, Germany). ^177^Lu was obtained from IDB Holland BV (Baarle-Nassau, Netherlands). For 6 GBq of ^177^Lu, 150 μg (143 nmol) of PSMA-617 was used for labeling. Yield and purity of the radio tracer were ≥99%. The activities were individually adjusted to the specific characteristics of the patient, such as body surface area, extent of tumor burden, bone marrow involvement, renal function, and dynamics of tumor progression. Thirty minutes prior treatment infusion, each patient received an intravenous hydration by 500 mL 0.9% NaCl and a cooling of the salivary glands. [^177^Lu]Lu-PSMA-617 was then infused intravenously over a period of 1 h.

### Response evaluation

**Fig. 2:**
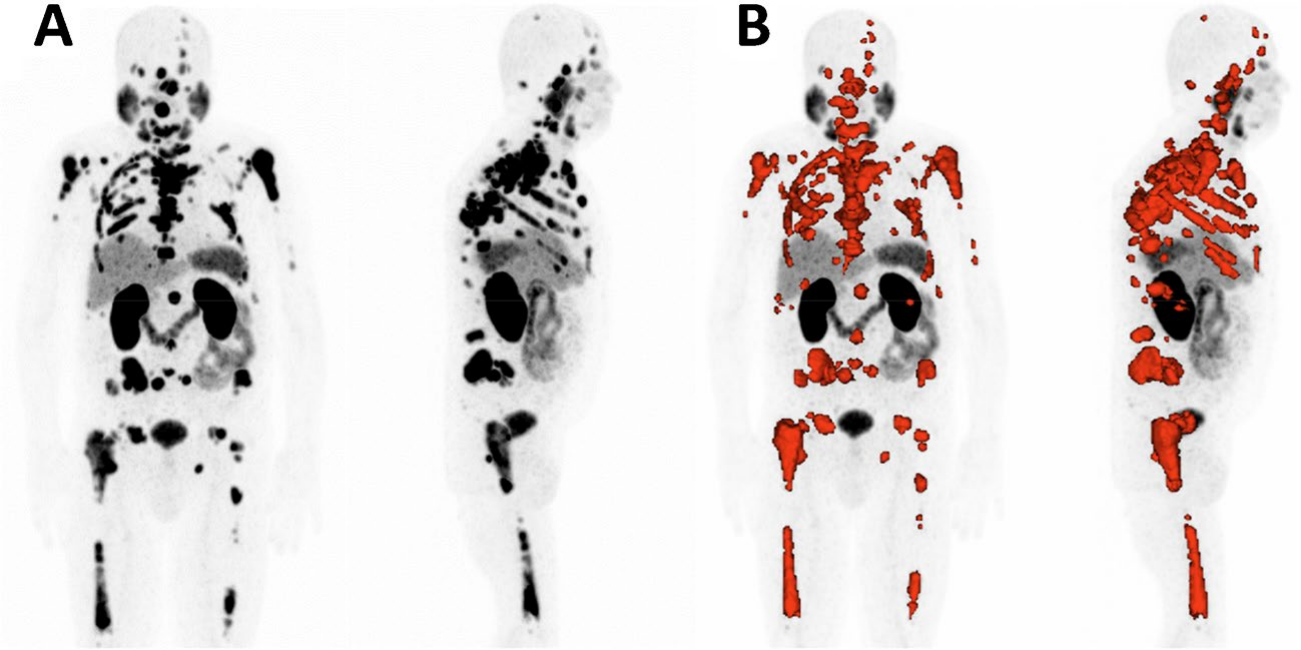
Representative semi-automated delineation of tumor burden using Syngo.Via. **A** Maximum intensity projection of [^68^Ga]Ga-PSMA-11 PET/CT. **B** The PSMA-positive tumor volume is delineated in red

For both [^68^Ga]Ga-PSMA-11 PET/CT scans, TLP was measured by the semi-automated tumor segmentation algorithm using Syngo.Via software (Enterprise VB 40B, Siemens, Erlangen, Germany). For delineation, standardized uptake of SUV ≥ 3 was used as a threshold, as described by Ferdinandus et al. [31]. Lesions with a volume < 0.2 mL were excluded. Physiological uptake such as in the liver, spleen, bladder, or salivary glands was manually excluded. Segmentation of liver metastases was performed according to our prior work using a threshold of 1.5 × SUV_mean_ of non-metastatic liver tissue. Exemplary tumor delineation in [^68^Ga]Ga-PSMA-11 PET/CT using Syngo.Via is depicted in Fig. 2.

TLP was defined as the summed products of volume and uptake (∑ Volume × SUV_mean_) of all lesions. To reflect the course of TLP value during therapy, ∆TLP was presented as a percentage value that is expressing the divergence of TLP values between baseline and follow-up [^68^Ga]Ga-PSMA-11 PET/CT (TLP_follow-up_/TLP_baseline_−1). Based on ΔTLP values, patients were classified in one out of three categories, which indicate therapy response: partial remission (PR), stable disease (SD), and progressive disease (PD). First, we assessed the most appropriate ∆TLP threshold values by comparison of Kaplan-Meier curves differing in applied thresholds. Subsequently, we analyzed the impact of occurrence of new metastasis, first (I), as an independent criterion for progression as it is recommended by the “PET response criteria in solid tumors 1.0” (PERCIST) [32], and second (II), the occurrence of new metastasis in combination with change in total tumor burden as recommended by “response evaluation criteria in prostate specific membrane antigen PET/CT 1.0” statement (RECIP1.0) [33]. In the latter, we followed the cut-off values and the consideration rules of new metastases for progression, stable disease, and partial remission by the RECIP framework, i.e., we adapted the RECIP1.0 framework which is based on the total PSMA tumor volume to TLP. Here, progression was defined as TLP increase > 20% with concomitant occurrence of new metastases, partial remission as TLP decrease >30% without occurrence of new metastases, and stable disease in all other cases. Last (III), we analyzed the biochemical response following the progression criterion of the “prostate cancer working group 3” (PCWG3) [34] for PSA serum value with a PSA increase >25% equalling PD. Biochemical partial remission was defined as a PSA decrease >50% and stable disease in any other case.

### Statistical analysis

Statistical analysis was performed using Prism Version 8.2.0 (GraphPad Software, San Diego, USA) and SPSS version 29 (IBM Corp., Armonk, USA). A *p* value of <0.05 was regarded as statistically significant. Data regarding OS was acquired from the regularly updated prospective registry (REALITY Study, NCT04833517). Start point of the period measuring OS was the date of first PSMA-RLT cycle. Endpoint of this study was either death or last contact to the subject. End date of follow-up was 2 May 2023. For comparisons of OS, we performed Kaplan-Meier curves and applied a log-rank test.

## Results

**Fig. 3:**
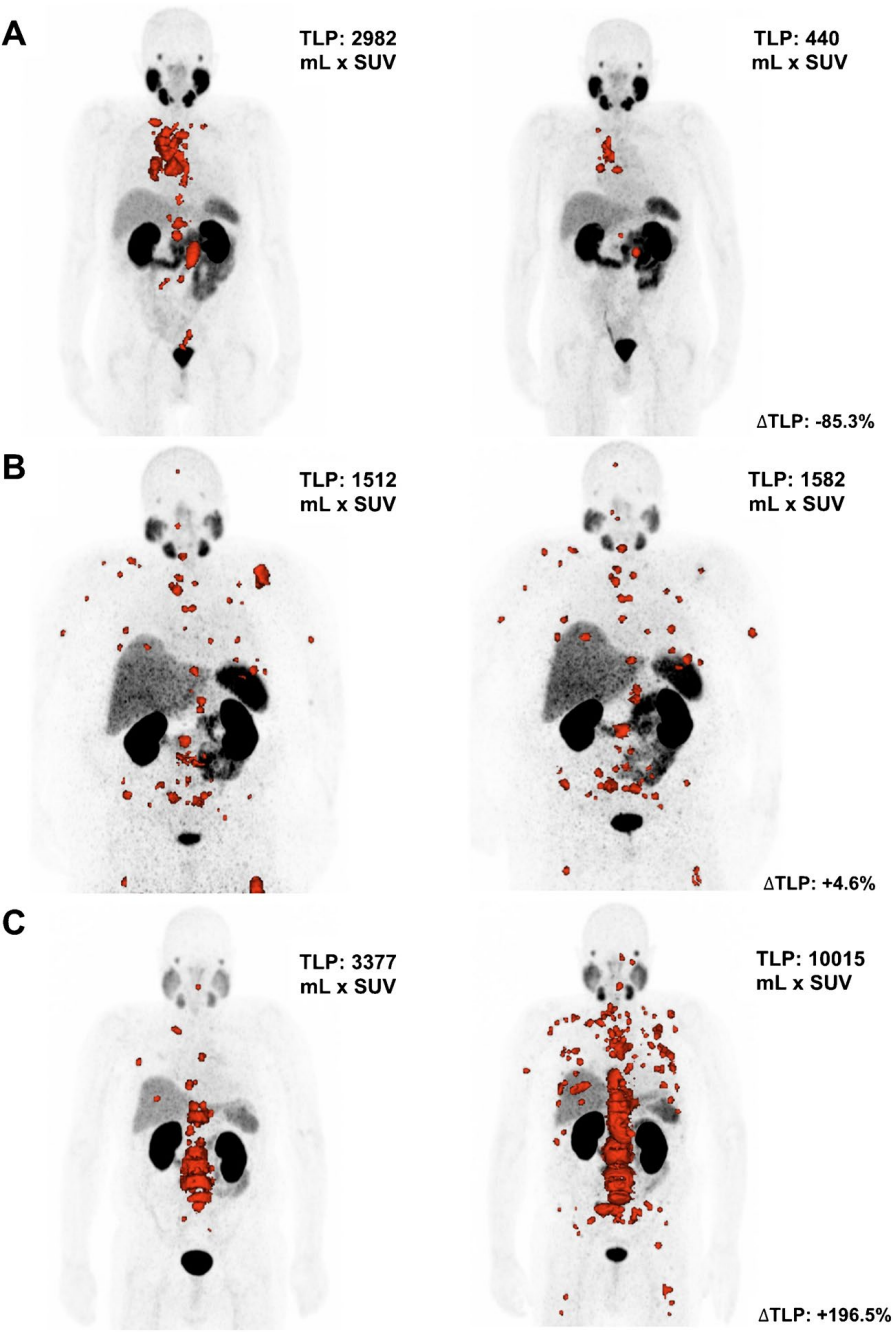
Change in tumor burden in three different patients after two cycles PSMA-RLT. Left side presents time-point at baseline, right side follow-up [^68^Ga]Ga-PSMA-11 PET/CT. Representative image of a patient **A** with decrease in TLP from 2982 mL × SUV at baseline to 440 mL × SUV at follow-up PET/CT (∆TLP: −85.3%; OS censored 8.8 m, alive after cut-off date), **B** presenting a steady TLP of 1512 and 1582 mL × SUV, respectively (∆TLP: +4.6%; OS 24.8 m), **C** showing a TLP value increasing from 3377 mL × SUV at baseline to 10,015 mL × SUV at follow-up PET/CT (∆TLP: +196.6%; OS 8.1 m)

Calculating median values for TLP demonstrated a decrease from pre-therapeutic 5711 mL × SUV (range: 127.6–38,638 mL × SUV) to post-therapeutic 2253 mL × SUV (range: 39.8–39,646 mL × SUV). The median change in TLP was −45.4% after two cycles of [^177^Lu]Lu-PSMA-617 RLT (range: −98.9–196.5%). Fig. 3 depicts three patient examples with either decreasing, increasing, or similar TLP after two cycles. The pre-therapeutic median PSA level was 130.0 ng/mL (range 2.9–9579 ng/mL). After two cycles of [^177^Lu]Lu-PSMA-617 RLT, median PSA level was 53.2 ng/mL (range 0.3–1083 ng/mL). Comparing pre- to post-therapy state, the median change of PSA was −56.8% (range −99.7–206.7 %).

**Fig. 4:**
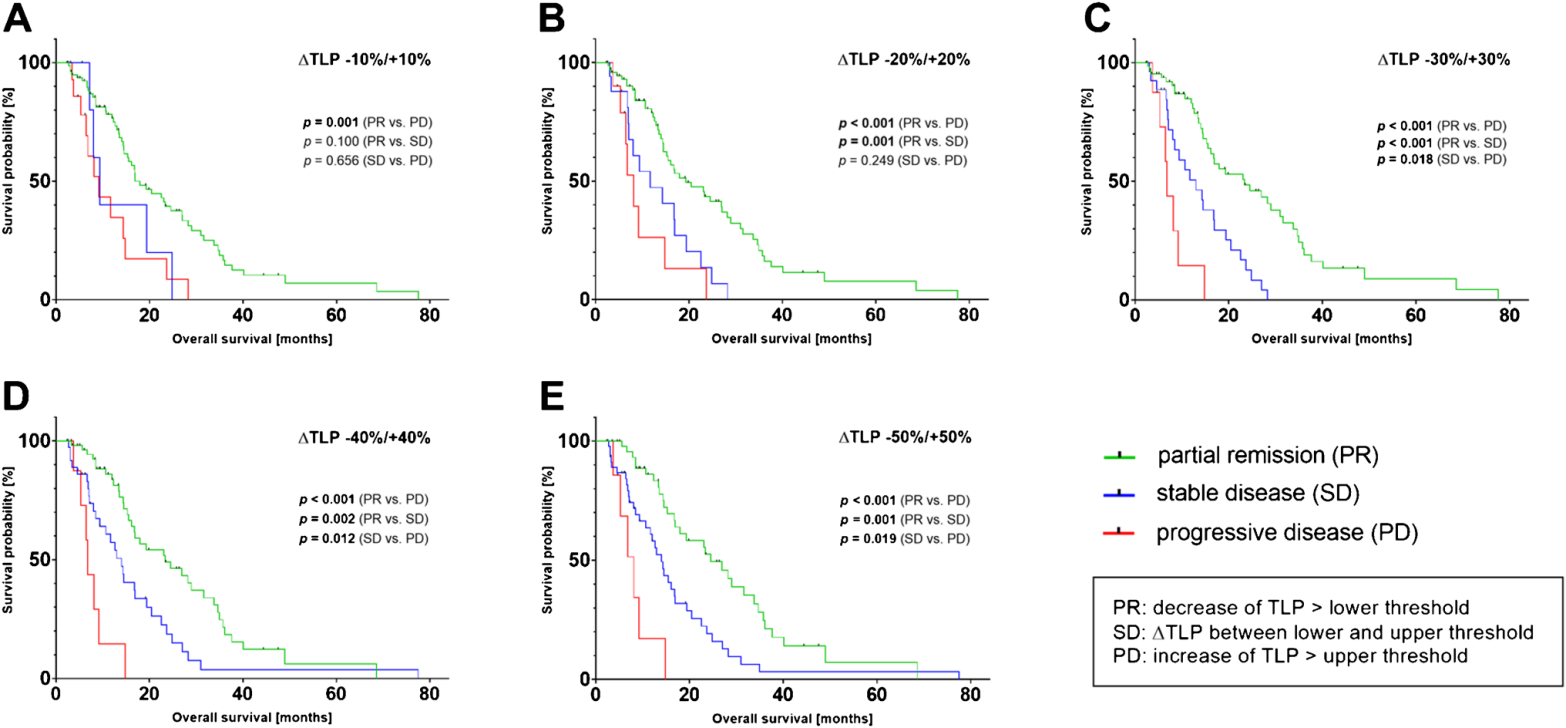
Kaplan-Meier curves for overall survival (OS) with categorization in partial remission (PR), stable disease (SD), or progressive disease (PD) using different thresholds of ∆TLP starting with *−*10%/+10% to *−*50%/+50% (**A**–**E**)

Patients were categorized as either PR, SD, or PD, depending on ∆TLP value. To evaluate the best ∆TLP threshold values for this categorization, multiple Kaplan-Meier curves with thresholds ranging from −10%/+10% to −50%/+50% were generated and are shown in Fig. 4. Table 2 summarizes data of OS for all groups.

**Table 2:** Statistics of overall survival (OS)

Group		*n*	Median OS (m)	95% CI lower threshold	95% CI upper threshold	Group comparison	*p* value(log-rank test)
Overall		102	16.8	13.6	19.9	-	-
∆TLP −10%/10%	PR	80	16.9	12.5	21.4	PR vs. PD	**0.001**
	SD	8	9.4	6.5	12.2	PR vs. SD	0.100
	PD	14	9.2	5.3	13.1	SD vs. PD	0.656
∆TLP −20%/+20%	PR	74	20.4	11.9	26.7	PR vs. PD	**0.001**
	SD	18	11.6	3.8	19.5	PR vs. SD	**0.001**
	PD	10	8.1	5.9	10.3	SD vs. PD	0.249
∆TLP −30%/+30%	PR	66	23.3	12.7	33.9	PR vs. PD	**<0.001**
	SD	28	13.0	7.1	18.8	PR vs. SD	**<0.001**
	PD	8	6.8	5.9	7.7	SD vs. PD	**0.018**
∆TLP −40%/+40%	PR	56	23.3	13.2	33.5	PR vs. PD	**<0.001**
	SD	38	14.0	10.6	17.5	PR vs. SD	**0.001**
	PD	8	6.8	5.9	7.7	SD vs. PD	**0.016**
∆TLP −50%/+50%	PR	47	24.5	18.1	31.0	PR vs. PD	**<0.001**
	SD	48	14.0	11.5	16.6	PR vs. SD	**0.001**
	PD	7	8.1	5.0	11.3	SD vs. PD	**0.019**
∆TLP −30%/+30% + NM as PD	PR	62	24.5	13.8	35.2	PR vs. PD	**<0.001**
	SD	19	13.0	6.4	19.6	PR vs. SD	**<0.001**
	PD	21	8.5	5.5	11.4	SD vs. PD	0.455
∆TLP −30%/+20% combined with NM criterion	PR	62	24.5	13.8	35.3	PR vs. PD	**<0.001**
	SD	30	11.7	5.6	17.8	PR vs. SD	**<0.001**
	PD	10	8.1	5.9	10.3	SD vs. PD	0.191
∆PSA −50%/+25%	PR	60	23.3	14.5	32.1	PR vs. PD	**0.001**
	SD	27	11.7	5.5	17.8	PR vs. SD	**0.002**
	PD	15	13.4	3.5	23.3	SD vs. PD	0.973

*p*-values reaching level of significance in bold

*NM* new metastases, *SD* stable disease, *PD* progressive disease, *PR* partial remission

Median overall survival (OS) including the entire patient cohort was 16.8 m (95% CI 13.6–19.9 m), median follow-up time of censored *n* = 29 cases was 9.2 m (minimum-maximum: 2.3–47.6 m). Using the lowest ∆TLP threshold value of ±10% (Fig. 4A), no significant differences in OS emerged between the categories PR and SD (*p* = 0.100) or between the categories of SD and PD (*p* = 0.656). Significant difference was only found between PR and PD (*p* = 0.001). When applying ∆TLP threshold values of −20%/+20% (Fig. 4B), no significant difference in OS was found comparing SD and PD (*p* = 0.249). However, significant differences were found comparing the groups of PR to SD (*p* = 0.001) as well as PR and PD (*p* < 0.001). For the thresholds −30%/+30% (Fig. 4C), significant differences between all response categories became apparent (PR/PD: *p* = 0.001; PR/SD: *p* = 0.001; SD/PD: *p* = 0.018). Likewise, applying thresholds of −40%/+40% (Fig. 4D) provides significant differences between all response groups (PR/PD: *p* = 0.001; PR/SD: *p* = 0.002; SD/PD: *p* = 0.012). Using thresholds of −50%/+50% (Fig. 4E) also showed significant differences between the three groups (PR/PD: *p* = 0.001; PR/SD: *p* = 0.001; SD/PD: *p* = 0.019).

**Fig. 5:**
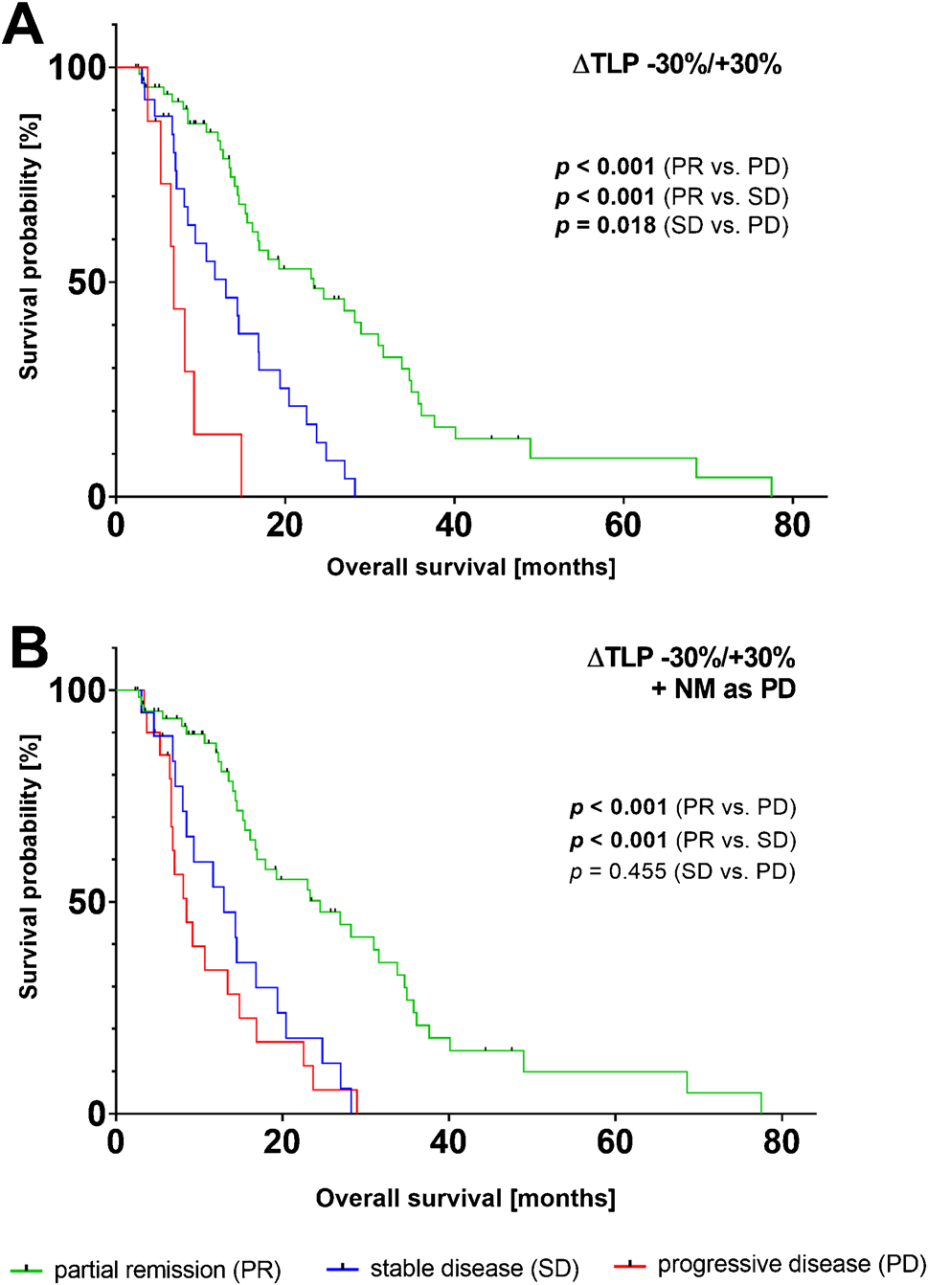
Impact of appearance of new metastases. Kaplan-Meier curves for overall survival (OS) with categorization in partial remission (PR), stable disease (SD), or progressive disease (PD) by **A** exclusively applying a ∆TLP threshold values of *−*30%/+30% for categorization and **B** adding the criterion of new metastases equaling PD to the criterion of ∆TLP *−*30%/+30%

On the follow-up PSMA PET/CT imaging after 2 cycles of PSMA-RLT 22/102 (21.6%), patients revealed new metastases (mostly bone, followed by lymph nodes, liver and lung metastases). Applying ∆TLP −30%/+30% with the addition that new occurrence of metastases results in PD (independent from TLP change), the significant difference between PR and PD remained present (*p* = 0.001, Fig. 5). Also, the significance distinguishing PR and SD (*p* = 0.001) was maintained. However, adding the new metastasis criterion resulted in loss of significance regarding the difference between SD and PD (*p* = 0.455).

**Fig. 6:**
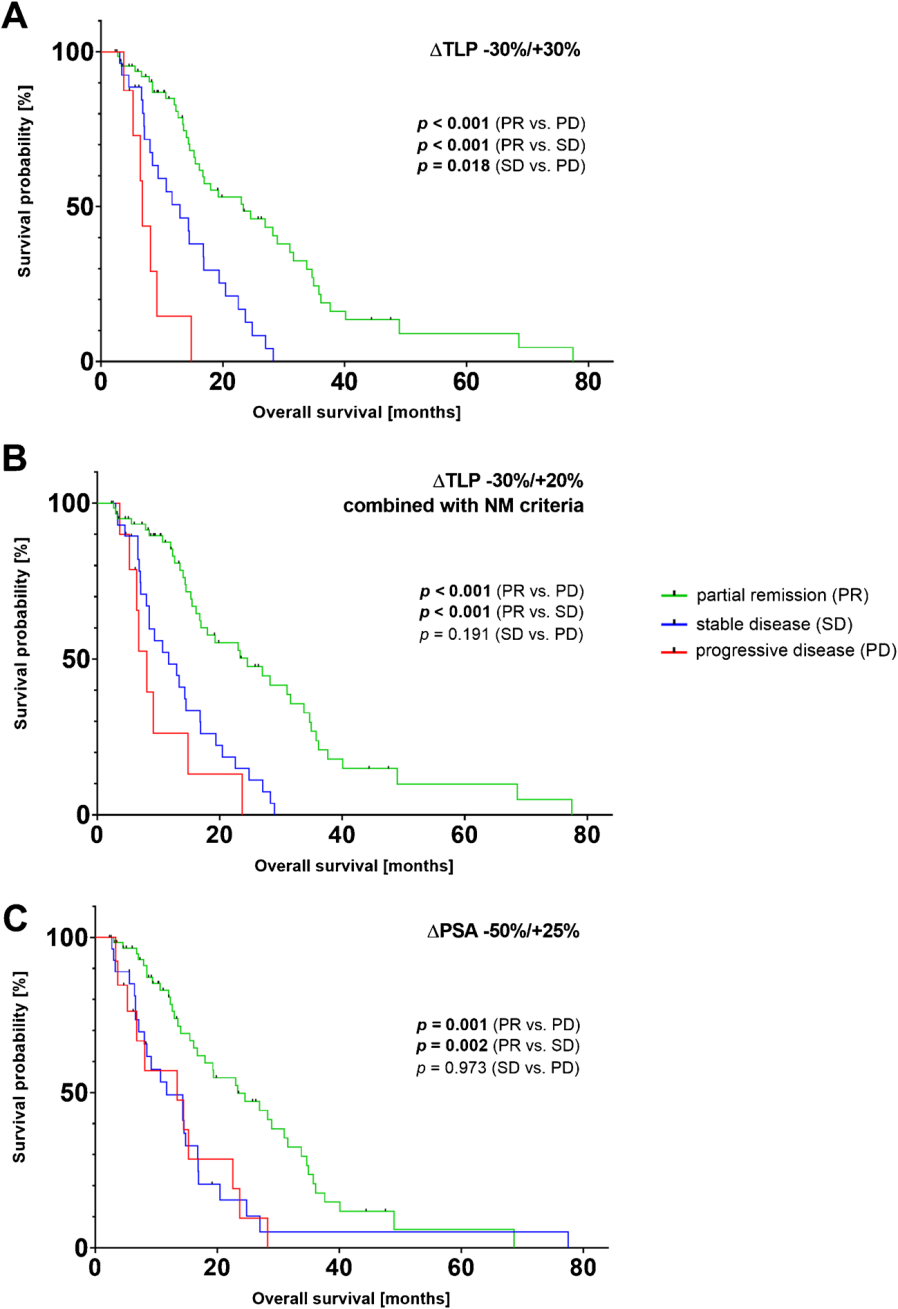
Comparison of three different response criteria. Kaplan-Meier curves for overall survival (OS) with categorization in partial remission (PR), stable disease (SD), or progressive disease (PD) by applying **A** ∆TLP threshold values of *−*30%/+30%, **B** ∆TLP *−*30%/+20% in combination with occurrence of new metastases (not as an independent criterion for progression, but as a mandatory criterion of progression and the absence of new metastases as a mandatory criterion of regression), and **C** ∆PSA −50%/+25% criteria

A comparison of categorization with ∆TLP *−*30%/+20% in combination with the occurrence of new metastases (not as an independent criterion, but as a mandatory criterion of progression and the absence of new metastases as a mandatory criterion of regression) and with ∆PSA *−*50%/+25% criterion is shown in Fig. 6. In contrast to categorization by ∆TLP thresholds *−*30%/+30%, where all response groups were significantly different in terms of OS, this was not found for all comparisons using the both mentioned criteria. Using ∆TLP *−*30%/+20% in combination with occurrence of new metastases, the categories PR and PD (*p* = 0.001) as well as response categories PR and SD (*p* = 0.001) showed significantly differences in OS. However, the response categories SD and PD presented no significant difference (*p* = 0.191). Similarly, using the change in serum PSA as a response criterion, PR vs. PD (*p* = 0.001) and PR vs. SD (*p* = 0.002) showed significant differences in OS. In contrast, SD and PD did not show significant difference (*p* = 0.973).

## Discussion

This study’s objective was to assess TLP as a biomarker for response evaluation in a prospective registry setting with a total of 102 patients, representing the largest monocentric study to date. Similar to the established TLG in [^18^F]FDG PET/CT, TLP is used to assess whole body tumor burden [26, 35]. In this study, we demonstrated that the change in TLP is a qualified criterion for response assessment in mCRPC patients undergoing [^177^Lu]Lu-PSMA-617 RLT. Our data suggest that −30%/+30% are appropriate thresholds to significantly distinguish PR, SD, and PD with respect to OS, whereas this differentiation was not completely achieved with other established criteria.

Our results are in line with our previous work in smaller cohort of patients, showing a strong association between change in TLP and OS [26]. The size of the present cohort allowed for threshold analyses for the determination of an appropriate threshold. For both −30%/+30% and higher thresholds such as −40%/+40% or −50%/+50%, OS differs significantly between PR, SD, and PD. The lowest of the cut-offs mentioned, −30%/+30%, seems to be the most appropriate and sensitive threshold to identify as many patients at risk or with a good prognosis as possible. The proposed −30%/+30% thresholds are consistent with the recommendations of the widely used PERCIST 1.0 framework, which refers to [^18^F]FDG PET/CT and the corresponding parameter TLG [32]. However, based on our data, the defining appearance of new lesions as PD, as known from PERCIST 1.0 or other criteria [32, 36], does not add additional value for risk assessment derived from TLP-dependent response in multi-metastatic CRPC patients. Significant differentiation of OS for SD and PD was not achieved by a combined criterion of change in tumor burden and occurrence of new metastases (as proposed by RECIP 1.0) [33]. Change in PSA value also failed to differentiate OS for SD and PD patients in our cohort. In contrast, the sole change in TLP with thresholds −30%/+30% was able to significantly discriminate OS between all groups, including SD and PD, and therefore seems to be preferable for response assessment.

In addition, the PSMA PET progression (PPP) criteria proposed by Fanti et al. exclusively distinguish between progress and non-progress, which does not explicitly capture the stable course of the disease [37]. A reliable differentiation not only between responders (PR) and non-responders (SD+PD) or between progression (PD) and no progression (PR+SD) but also between SD and PD is important to allow better individualized adaptation of treatment. Such an adjustment may be, for example, a change or augmentation with the alpha emitter ^225^Ac [38–40], a combination with another, e.g., androgen-axis drugs or immunotherapy [41, 42], or a switch to a completely different therapy.

The assessment of TLP has multiple advantages over target lesion–based assessments. In contrast to the evaluation and determination of target lesions, all metastases are quantitatively included, and high reproducibility as well as inter-reader agreement of TLP is expected due to the semi-automated algorithm. However, clinical feasibility may be challenged by the time required for the procedure (approx. 20–30 min per patient), but further improvement of automated segmentation software through artificial intelligence may facilitate clinical implementation.

Based on our results, molecular imaging response assessment derived from PSMA PET/CT using the parameter TLP is a qualified monitoring tool for mCRPC patients undergoing PSMA-RLT. Broader use of TLP, such as in the setting of other therapies and other stages of prostate cancer, may be both applicable and beneficial. Future studies, ideally in prospective setting, are recommended.

The data presented herein is subject to some limitations. First, the study is limited due to its retrospective nature. Although the present study reports on a larger number of patients than our previous study, larger cohorts are still needed to confirm the results. It should also be noted that the cohort in this study was pre-selected and the results are limited according to the inclusion criteria of the study. A possible selection bias cannot be excluded. Therefore, a non-pre-selected study seems necessary to extrapolate our findings to a broader spectrum of patients with PC. A potential bias could also arise from the application of individually adjusted activities. In addition, it should be noted that there are different methods and definitions for TLP, which may lead to different results [43–45]. We used the method by Ferdinandus et al., which uses an absolute value to segment SUV ≥ 3 [31]. Relative thresholds (e.g., 41% or 50%), as recommended for TLG [46], are also possible and further studies are required to address this.

## Conclusion

In the largest monocentric study to date, the whole-body parameter TLP, derived by PSMA-PET/CT and reflecting the total viable tumor burden, is shown to be a prognostic PET biomarker for mCRPC patients undergoing [^177^Lu]Lu-PSMA-617 RLT, with its change after 2 treatment cycles predicting overall survival. ∆TLP thresholds of −30%/+30% were found to be the most appropriate for significantly differentiating between PR, SD, and PD. Notably, it differentiated between SD and PD while other established criteria did not. In the presence of this whole-body tumor burden biomarker TLP, the development of new metastases is not a required information for predicting survival in multi-metastatic CRPC patients.

## Data Availability

The datasets used and analyzed during the current study are available from the corresponding author on reasonable request. Data of *n* = 66 patients included in this study have been previously published [26].
